# Editorial: Evidence for Assessing Drug Safety and Drug Use in Older People

**DOI:** 10.3389/fphar.2022.941813

**Published:** 2022-06-01

**Authors:** Luciane Cruz Lopes, Ria Benko, Marcio Galvão Oliveira, Vera Maria Vieira Paniz, Brian Godman, Fabiane Raquel Motter

**Affiliations:** ^1^ Graduate Course in Pharmaceutical Sciences, University of Sorocaba (Uniso), São Paulo, Brazil; ^2^ Institution of Clinical Pharmacy, Faculty of Pharmacy, University of Szeged, Szeged, Hungary; ^3^ Central Pharmacy, Albert Szent Györgyi Medical Center, University of Szeged, Szeged, Hungary; ^4^ Department of Emergency Medicine, Albert Szent Györgyi Medical Center, University of Szeged, Szeged, Hungary; ^5^ Multidisciplinary Health Institute, Federal University of Bahia, Vitória da Conquista, Brazil; ^6^ Postgraduate Program in Collective Health, University of Vale do Rio dos Sinos (UNISINOS), São Leopoldo, Brazil; ^7^ Strathclyde Institute of Pharmacy and Biomedical Sciences, Faculty of Science, University of Strathclyde, Glasgow, United Kingdom; ^8^ Centre of Medical and Bio Allied Health Sciences Research, Ajman University, Ajman, United Arab Emirates; ^9^ Division of Public Health Pharmacy and Management, School of Pharmacy, Sefako Makgatho Health Sciences University, Pretoria, South Africa

**Keywords:** drug safety, adverse drug reactions, medication without harm, older patients, deprescribing, potentially inappropriate medications

Prescribing for older patients presents several challenges. Older people often suffer from two or more chronic diseases (multimorbidity) and therefore use a greater number of medications compared to other age groups. As a result, they are more susceptible polypharmacy, and associated drug-related problems, including potentially inappropriate medication (PIM), drug ineffectiveness, drug interactions, and adverse drug events (Nobili et al., 2011; Aggarwal et al., 2020). Consequently, optimizing drug therapy is a crucial part of caring for an elderly individual. This is increasingly important given the rising number of older adults across countries in the coming years, with one in six of the world’s population over 60 by 2050 and the associated resource implications (World Health Organization, 2021).

Many studies (Oliveira et al., 2012; Shah and Hajjar, 2012; Khatter et al., 2021; Xu et al., 2021) point out that polypharmacy is a risk factor for PIM, particularly for older patients. The more medications a patient are taking, the more likely they are to have an adverse drug event (ADE), potentially experience a drug-drug interaction, take a PIM, or be non-compliant to one or more of the medications prescribed (Shah and Hajjar, 2012).

This Research Topic included 23 articles and nine of them (Ambrož et al.; Candeias et al.; Chen et al.; Kardas et al.; Kurczewska-Michalak et al.; Machado-Duque et al.; Perpétuo et al.; Schneider et al.; Bobrova et al.) studied PIM and polypharmacy in older adults. Four of them (Kardas et al.; Khatter et al., 2021; Machado-Duque et al.; Schneider et al.) estimate the prevalence of PIM or polypharmacy in older adults.

In recent years, several strategies and tools have been developed to identify the inappropriate prescribing of medications. Typically, adaptations and selections have to be made depending on the setting and the medications available in a country (Motter et al., 2018; Motter et al., 2019). STOPP (Screening Tool of Older Persons’ Prescriptions) and START (Screening Tool to Alert to Right Treatment) are criteria typically used as a tool for clinicians to review PIMs in older adults and have been endorsed as best practice by some organizations. The study of Bobrova et al. developed an integrated PIM clinical decision support tool for identification of drug-related problems among geriatric patients in geriatric multi-morbid polypharmacy patients, using the EU-PIM and EURO-FORTA lists, with a focus on high-risk medications. The articles from Candeias et al. and Perpétuo et al. analyzed the concordance and prevalence of PIM different tools.

In particular, polypharmacy is known to cause a higher risk of ADEs as well as drug-drug interactions, which often leads to poor compliance with prescribed medicines. All these negatively impact on the health of patients as well as increase the risk of geriatric syndromes, e.g., cognitive impairment or falls. An important disparity is the difference of sex and gender in the proportion of types of medication used among older patients Lu et al., which needs to be factored into future prescribing.

Avoidable ADEs are the consequences of inappropriate drug prescribing including inappropriate polypharmacy. This, in turn, leads to increased costs and health care expenditures (Maher et al., 2014). The studies of Alnijadi et al. and Katsuno et al. analyzed the direct cost of managing adverse drug events and that of avoidable ADEs as well as cost-related medication non-compliance with medicines on healthcare utilization and patient-reported outcomes. Consequently, we are seeing health authorities across countries instigate activities to improve prescribing in the older adults and reduce ADEs and their associated costs, with these activities likely to grow with an increasing older population (MacBride-Stewart et al., 2021).

Numerous factors contribute to the appropriateness and comprehensive quality of drug prescribing. The process of prescribing a medication is multifaceted and includes: verifying that a drug is indicated and avoiding overuse of medicines for prevention, selecting the best drug, determining a dose and duration appropriate for the patient’s physiologic status, monitoring for effectiveness and toxicity, educating the patient about expected side effects, and indications for seeking a consultation.

Zazzara et al. verified the medication use and costs among older adults aged 90 years and conclude that the persistent use of preventive medications highlights the potential lack of awareness regarding medication rationalization among clinicians and provided guidance for optimizing prescriptions. Chen et al. identified factors that have an impact on the management of potentially inappropriate prescribing and concluded that gerontology practitioners should be prudent in applying clinical guidelines to provide personalized, comprehensive assessment of decision making of prescriptions, especially in socioeconomically deprived areas. Qu et al. explored the relationship between drug literacy and frailty and conclude that the first was an important consideration in the development, implementation, and evaluation of frailty.

Approaches to decrease inappropriate prescribing in older adults include educational interventions, peer comparison feedback, computerized order entry and decision support, multidisciplinary team care led by physicians, clinical pharmacists, and combinations of these approaches (Rochon, 2022). The scoping review of (Kurczewska-Michalak et al.) published in this Research Topic mapped available interventions and more complex strategies to prevent and manage polypharmacy in the older adults and discussed their potential implementation. The authors concluded that the development of strategies for the detection and prevention of drug-related problems is important to guide and support clinical decision-making and strengthen research into drug safety. This is an essential condition for achieving wide-ranging improvements in the management of older patients. Whilst different approaches have been identified to avoid drug-related problems in older patients, there is still insufficient information about their clinical importance or their public health impact. The authors also suggested that guidance on polypharmacy management in older adults is still limited. Initiatives to understand and conceptualize healthcare professional’s barriers and enablers can be used to increase knowledge translation and strengthen capacity for appropriate interventions in routine clinical practice (Motter et al., 2021).

This Research Topic also included studies comparing the efficacy and safety of anticoagulants or antiplatelets in cardiovascular disease (Wawruch et al.; Zhao et al.; Li et al.). This is important as there were concerns with excessive bleeding in the elderly when dabigatran, the first non-vitamin K antagonist oral anticoagulants (NOAC) was first launched (Malmström et al., 2013). Physician knowledge has now grown, with more recent studies comparing key issues such as effectiveness and safety among the NOACs (Mueller et al., 2019; Komen et al., 2021).

Studies that analyzed the safety and efficacy of medications in other common problems in older patients were also included in this Research Topic. Two studies (Huang et al.; Yang et al.) estimated the efficacy of propofol in adult or older patients with different conditions. Two systematics reviews (Huang et al.; Zhang et al.) studied the efficacy and safety of drug use in secondary care. Gao et al. conducted a network meta-analysis to summarize all available evidence about relative effectiveness of different pharmacotherapy of macular edema secondary to retinal vein occlusion. Yu et al. conducted a cross-sectional study, analyzing the trends in the topical prescription’s treatment of old patients with dry eye disease.

Optimizing the use of medications is increasingly recognized as an important pillar in the health care of older people. Collectively, this Research Topic highlights pertinent concerns related to the safe use of medications in this age group and promotes awareness of optimizing older adults’ medication regimens. The results demonstrate that improving the quality of medication use and medication safety are still important challenges for healthcare professionals who care for older patients. Other initiatives are required for this field to reach its full potential of optimizing drug use in older patient to improve their health care outcomes within available resources.
